# Cross-talk among HMGA1 and FoxO1 in control of nuclear insulin signaling

**DOI:** 10.1038/s41598-018-26968-3

**Published:** 2018-06-04

**Authors:** Eusebio Chiefari, Biagio Arcidiacono, Camillo Palmieri, Domenica Maria Corigliano, Valeria Maria Morittu, Domenico Britti, Michal Armoni, Daniela Patrizia Foti, Antonio Brunetti

**Affiliations:** 1 Department of Health Sciences, University of Catanzaro “Magna Græcia”, Catanzaro, Italy; 2 Department of Clinical and Experimental Medicine, University of Catanzaro “Magna Græcia”, Catanzaro, Italy; 3 Institute of Immunology and Tissue Typing, Rambam Medical Center, Haifa, Israel

## Abstract

As a mediator of insulin-regulated gene expression, the FoxO1 transcription factor represents a master regulator of liver glucose metabolism. We previously reported that the high-mobility group AT-hook 1 (HMGA1) protein, a molecular switch for the *insulin receptor* gene, functions also as a downstream target of the insulin receptor signaling pathway, representing a critical nuclear mediator of insulin function. Here, we investigated whether a functional relationship existed between FoxO1 and HMGA1, which might help explain insulin-mediated gene transcription in the liver. To this end, as a model study, we investigated the canonical FoxO1-HMGA1-responsive *IGFBP1* gene, whose hepatic expression is regulated by insulin. By using a conventional GST-pull down assay combined with co-immunoprecipitation and Fluorescence Resonance Energy Transfer (FRET) analyses, we provide evidence of a physical interaction between FoxO1 and HMGA1. Further investigation with chromatin immunoprecipitation, confocal microscopy, and Fluorescence Recovery After Photobleaching (FRAP) technology indicated a functional significance of this interaction, in both basal and insulin-stimulated states, providing evidence that, by modulating FoxO1 transactivation, HMGA1 is essential for FoxO1-induced *IGFBP1* gene expression, and thereby a critical modulator of insulin-mediated *FoxO1* regulation in the liver. Collectively, our findings highlight a novel FoxO1/HMGA1-mediated mechanism by which insulin may regulate gene expression and metabolism.

## Introduction

The regulation of glucose metabolism and homeostasis is a central component of living systems. In mammals, this function is performed through distinct but interrelated cell signaling pathways that mediate the transduction of hormonal and nutrient stimuli to the nucleus, resulting in modifications of nuclear regulatory proteins (transcription factors), which bind to specific sites on the DNA and cause the activation or repression of genes and gene networks involved in these metabolic processes. Among the nuclear factors that act downstream of these pathways, the forkhead box protein O1 (FoxO1) is an important nutrient-sensing transcription factor that modulates the expression and activity of insulin-sensitive genes involved in gluconeogenesis, glycogenolysis and energy homeostasis^1–3^. Central to the functional regulation of FoxO1 is its nucleocytoplasmic shuttling following FoxO1 protein phosphorylation by the insulin-dependent phosphatidylinositol 3-Kinase/Akt (PI3K/Akt) signaling pathway^4–8^, a process that is further enhanced by FoxO1 acetylation^9^. Thus, in fasting conditions when insulin is low and the PI3K/Akt pathway is abrogated, FoxO1 binds the gluconeogenic genes^10–13^, and activates their expression, contributing to the maintenance of fasting euglycemia. Vice versa, in fed conditions, phosphorylation of FoxO1 by insulin, leading to the detachment of FoxO1 from DNA and its shuttling into the cytosol, represses gluconeogenesis^4,14–18^, thereby contributing to the maintenance of postprandial glucose homeostasis.

The high-mobility group A1 (HMGA1) protein is an architectural factor that binds to adenine-thymine (A-T) rich regions of DNA^19^. By itself, HMGA1 has no intrinsic transcriptional activity; rather, it can transactivate promoters by facilitating the assembly and stability of higher-order transcriptional complexes – so-called enhanceosomes – that drive gene transcription in response to extracellular and intracellular signals^20,21^. Such signals may affect HMGA1 function by inducing changes in post-translational protein modifications that markedly influence HMGA1 ability to interact with DNA substrates, other proteins and chromatin^21,22^. Previous studies from our laboratory have demonstrated that HMGA1 functions as a downstream nuclear target of the insulin receptor signaling pathway *in vivo*^23^. In particular, we showed that the hypoglycemic effect of insulin after meal, through the transcriptional repression of gluconeogenic genes, is mediated by HMGA1^23^. Insulin exerts this transcriptional repression by triggering HMGA1 phosphorylation at three serine residues, via a PI3K/Akt pathway, inducing HMGA1 detachment from gluconeogenic gene promoters^23^. On the other hand, a relationship between HMGA1 and the insulin receptor signaling system has been demonstrated before, showing that HMGA1 is a key regulator of the expression of the insulin receptor^24^. Consistently with these observations, *HMGA1* gene defects produce insulin resistance and type 2 diabetes in humans and mice^25–28^, whereas protection against insulin resistance has been reported in transgenic mice overexpressing Hmga1^29^.

Increasing evidences demonstrated that FoxO1 executes its function on gene promoters by interacting with other nuclear proteins with which it assembles different transcriptional complexes^13,30–33^. However, the mechanisms by which these processes regulate FoxO1 activity are still largely unknown. On the base of the above considerations, here we explored the hypothesis that the insulin-mediated FoxO1-regulated metabolic pathway in the liver could be modulated by direct interactions of HMGA1 with FoxO1.

## Results

### Physical interaction between HMGA1 and FoxO1

**Figure 1:**
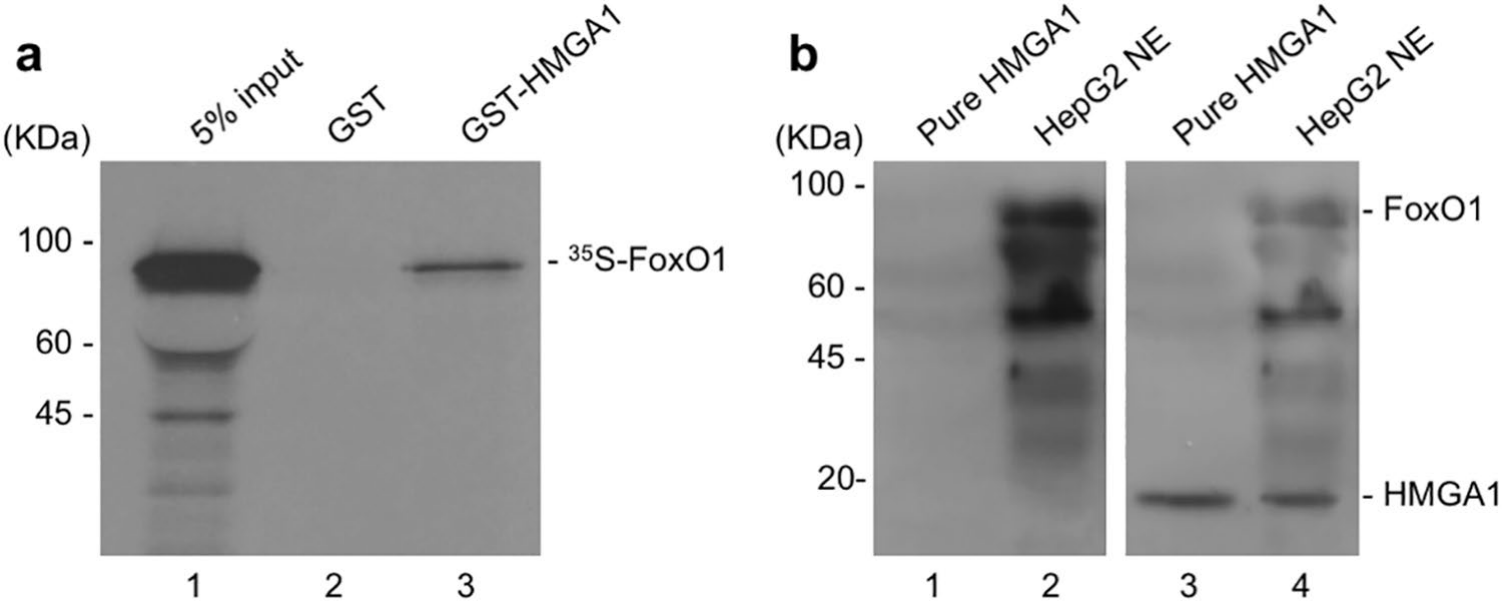
Physical association between HMGA1 and FoxO1. (**a**) SDS-PAGE of ^35^S-FoxO1a bound to GST-HMGA1 resin. Lane 1, input; Lane 2, GST alone; Lane 3, labeled protein retained by GST-HMGA1 resin. (**b**) Immunoprecipitation (IP) of FoxO1 and HMGA1 by using the anti-HMGA1 antibody followed by immunoblotting with the anti-FoxO1 antibody (lanes 1-2), or the anti-HMGA1 antibody (lanes 3-4), after reprobing the same transfer. Lanes 1 and 3, 10 ng of pure HMGA1; lanes 2 and 4, HepG2 nuclear extract (NE; 500 µg). Lanes 1 and 3, protein was directly applied to the gel without binding to and elution from protein A beads. Cropped blots are shown in the figures. Full-length blots are presented in Supplementary Fig. S1.

We previously showed that phosphorylation of HMGA1 represents a critical event in mediating the insulin’s effect on gluconeogenic genes *PEPCK* and *G6Pase*, as well as other insulin target genes, such as the *insulin receptor* and the *IGFBP1* genes^23^. On the other hand, FoxO1 is an important mediator of insulin action at these levels^34,35^, and cytoplasmic retention of FoxO1 via PI3K/AKT phosphorylation is considered to be a mechanism of insulin-mediated repression of insulin target genes^34,35^. Thus, the possibility that an interplay among HMGA1 and FoxO1 could be a fundamental prerequisite for these functions was investigated in this study. First, we tested the possibility that HMGA1 could physically interact with FoxO1, in the absence of DNA. We performed a GST pull-down assay, in which a GST-HMGA1 affinity resin was analyzed for its ability to specifically retain *in vitro*-translated ^35^S-labeled FoxO1. As shown in Fig. 1a, FoxO1 was retained by GST-HMGA1 but not GST alone, indicating that physical interaction between FoxO1 and HMGA1 occurs *in vitro*, in the absence of chromatin/DNA. The interaction between HMGA1 and FoxO1 was investigated further in co-immunoprecipitation studies with nuclear extracts from HepG2 cells, a cell line naturally expressing both HMGA1 and FoxO1 proteins, in the presence of an anti-HMGA1 specific antibody immobilized on protein A beads. As shown in Fig. 1b, immunoprecipitation of HMGA1 from HepG2 nuclear extracts, followed by western blot analysis for FoxO1, revealed a higher MW band (80-kDa), which migrated in a position corresponding to the size of FoxO1. When the same transfer was reprobed with an anti-HMGA1 specific antibody, a low MW band (12-kDa), which migrated in a position corresponding to the size of pure HMGA1, was detected as well (Fig. 1b). Co-immunoprecipitation between HMGA1 and FoxO1 was also observed *in vivo*, in liver extracts from wild-type mice (not shown), in which, however, specific signals on immunoblots were qualitatively poor as compared to those from HepG2 cells.

Thus, these results clearly indicate that HMGA1 and FoxO1 physically interact with each other *in vitro*, in cell free extracts, as well as in the context of intact cells and liver tissue samples.

### Subcellular colocalization of HMGA1 and FoxO1

**Figure 2:**
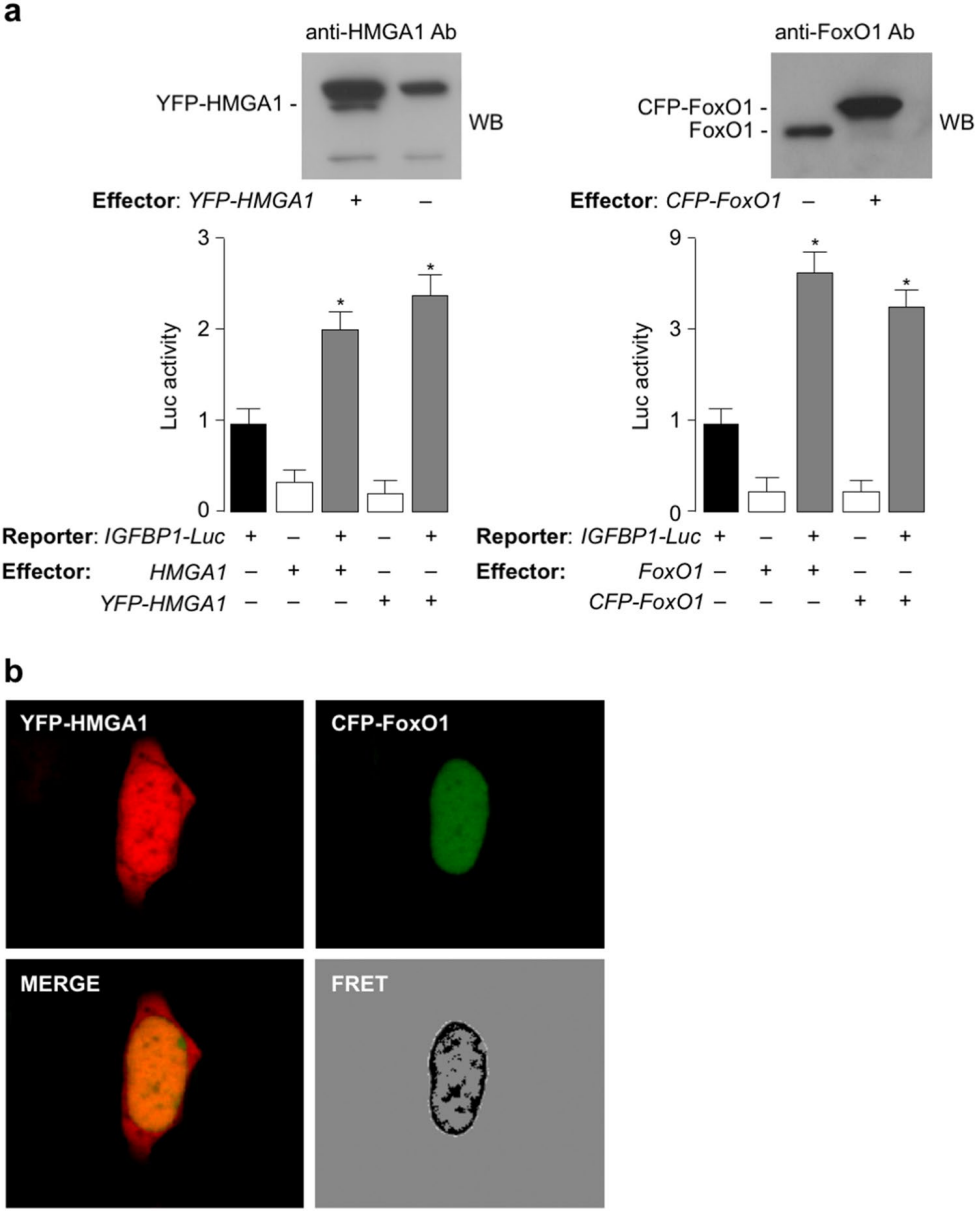
HMGA1 and FoxO1 fusion protein expression and *IGFBP1*-Luc reporter gene assay following transfection of effector vectors for YFP-HMGA1 and CFP-FoxO1 into HEK-293 cells. (**a**) Representative WBs of YFP-HMGA1 and CFP-FoxO1 fusion proteins are shown in the left and right upper panels of the figure, respectively. Functional activity of both fusion proteins is shown in reporter gene assays, using HEK-293 cells cotransfected with the *IGFBP1*-Luc reporter construct in the presence of either *HMGA1/YFP-HMGA1* (left) or *FoxO1/CFP-FoxO1* (right) effector vectors (0.1 µg each). Cropped blots are shown in the figure. Full-length WBs are presented in Supplementary Fig. S1. (**b**) FRET. Distribution of YFP-HMGA1, CFP-FoxO1, and FRET signal in starved HEK-293 cells. Distribution of maximum FRET signal is presented using a black-white lookup table.

FoxO1’s activity is finely regulated by two distinct mechanisms: by post-translational modifications, which drive subcellular localization of FoxO1, and by protein-protein interactions, that mediate transcriptional activation^36,37^. As previously reported, both mechanisms are triggered by a wide variety of extracellular stimuli, which include oxidative stress, growth factors, and nutrients^36–39^. To substantiate the interaction between HMGA1 and FoxO1, and to address their subcellular localization in living cells, we performed FRET studies that finely allow the detection of associated proteins in living cells in real time. To this end, the coding sequences of both HMGA1 (HMGA1a isoform) and FoxO1 (FoxO1a isoform) were tagged with the yellow fluorescent protein (YFP) and the cyan fluorescent protein (CFP), respectively, and expressed in HEK-293 cells that were deprived of serum (starved) to prevent nuclear/cytoplasmic shuttling of the FoxO1 protein. As determined by Western blot analysis and reporter gene assay, both fusion proteins were expressed and functionally active following transfection of expression plasmids into cells (Fig. 2a). As shown in Fig. 2b, YFP-HMGA1 and CFP-FoxO1 exhibited a robust FRET signal in the nucleus of serum starved HEK-293 cells, with an almost equivalent distribution between the heterochromatin chromatin domains, found near the nuclear envelope, and the more transcriptionally active central nuclear position, which supports the physical interaction between the two proteins at this level. HEK-293 cells were ideally suited for FRET experiments since they do not express appreciable levels of endogenous HMGA1 and FoxO1, a condition that improves the analysis of transfected fluorescent proteins.

### Functional significance of HMGA1-FoxO1 protein-protein interaction

**Figure 3:**
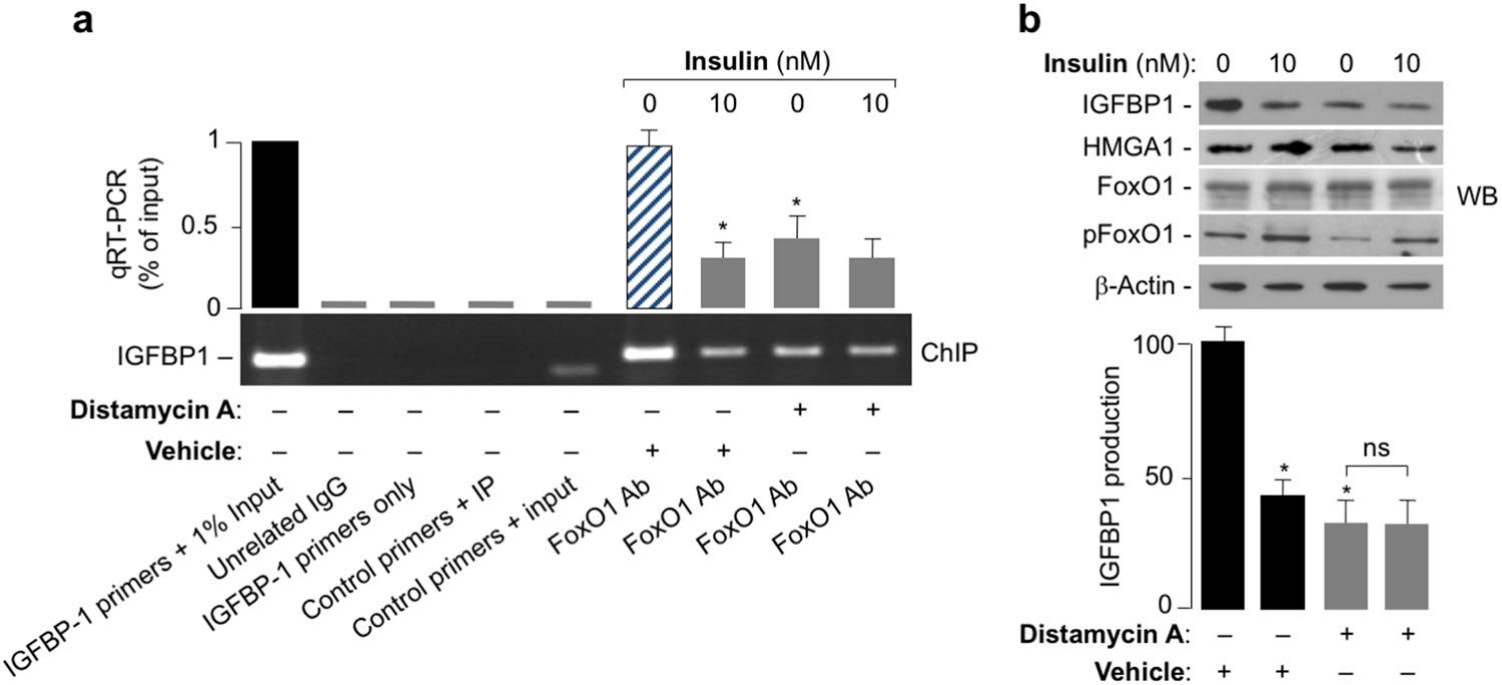
Functional significance of HMGA1/FoxO1 interaction. (**a**) Representative ChIP of the *IGFBP1* promoter gene with anti-FoxO1 antibody (Ab) and qRT-PCR of ChIP-ed samples in HepG2 cells untreated or pretreated with distamycin A (100–150 µM), in the absence or in the presence of 10 nM insulin. *P < 0.05 *vs* untreated cells (slashed bars). Cropped gel is shown in the figure. Full-length ChIP is presented in Supplementary Fig. S1. (**b**) IGFBP1 suppression and FoxO1 phosphorylation in HepG2 cells under the same conditions as in (**a**). Conditioned medium samples and cell nuclear extracts were collected after 12 h insulin treatment and assayed by western blot (WB) for IGFBP1, HMGA1, total FoxO1, and phosphorylated pFoxO1. Densitometer scanning of IGFBP1 is shown in bar graphs. Results are expressed as percentages of the IGFBP1 protein production as obtained in the presence of vehicle alone. Cropped blots are shown in the figure. Full-length WBs are presented in Supplementary Fig. S1. *P < 0.05 *vs* vehicle alone.

The functional significance of the interaction between HMGA1 and FoxO1 *in vivo* was then investigated at the level of the endogenous *IGFBP1* genomic locus, which is recognized as a downstream target of the insulin receptor signaling pathway, and whose transcriptional regulation by either FoxO1 or HMGA1 has also been reported^10,14,23,40^. Using the *IGFBP1* gene promoter as a target of insulin action, we performed ChIP assays in HepG2 cells, along with quantitative qRT-PCR of ChIP-ed samples. As shown in Fig. 3a, FoxO1 occupancy at the *IGFBP1* chromatin target site was downregulated by insulin and was significantly reduced in cells pretreated with distamycin A, a DNA-binding agent which selectively blocks DNA binding by HMGA1^24^. In line with our previous observations indicating that functional integrity of HMGA1 is required for normal insulin action^23^, insulin failed to downregulate FoxO1-DNA binding in the presence of distamycin A (Fig. 3a). DNA occupancy by FoxO1 at these sites closely paralleled the changes in IGFBP1 protein production in HepG2 cells under the same experimental conditions as those used for ChIP (Fig. 3b), thus suggesting that binding of HMGA1 to DNA is required for FoxO1-DNA interaction and transcriptional activity and may constitute a prerequisite for the functional regulation of FoxO1 by insulin.

**Figure 4:**
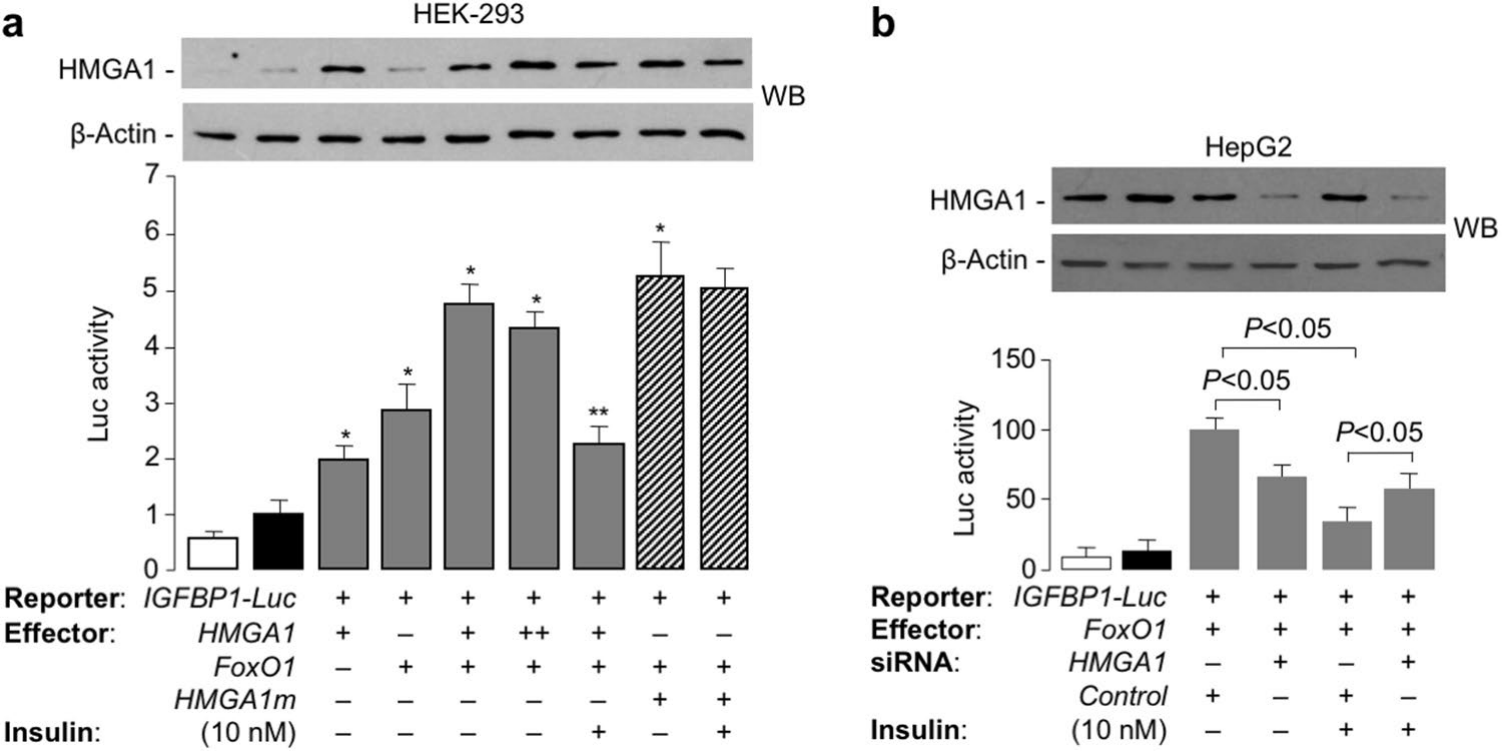
HMGA1/FoxO1 interaction and *IGFBP1* gene transcription. (**a**) HEK-293 cells, barely expressing endogenous HMGA1, were cotransfected with *IGFBP1*-Luc reporter vector, plus *FoxO1* expression plasmid (0.1 µg), either in the absence or presence of increasing amounts (0, 0.25, 1 µg) of effector vectors for HMGA1 wild-type (*HMGA1*, gray bars) or mutant HMGA1 (*HMGA1m*, slashed bars) (0.25 µg). At 48 h after transfection, cells were incubated in the absence (−) or presence (+) of insulin and cell lysates were prepared 4 h later. Cell lysates were divided into two aliquots; one was used for Luc activity, and the other for WB analysis as a control of HMGA1 protein expression. Values of Luc activity in each condition are expressed as factors by which reporter activity increased or decreased as compared to the level of Luc activity obtained in transfections with *IGFBP1*-Luc reporter vector alone (black column), which is assigned an arbitrary value of 1. White bar, pcDNA3 vector without an insert. Data represent the means ± s.e.m. for three separate experiments. Representative WBs of endogenous and overexpressed HMGA1a and HMGA1m proteins are shown. *P < 0.05 *vs* control (black bar); **P < 0.05 *vs* relative insulin-untreated cells. (**b**) Human *IGFBP1*-Luc reporter vector (2 µg) was cotransfected with 0.1 µg of *FoxO1* effector plasmid into HepG2 cells pretreated with anti-HMGA1 siRNA or a nontargeting control siRNA. At 48 h after transfection, cells were incubated in the absence (−) or presence (+) of insulin, cell lysates were prepared 4 h later, and Luc activity was measured. Data represent the means ± s.e.m. for three separate experiments; Luc activity in each condition is expressed as a percentage of the reporter activity obtained in transfections with *IGFBP1*-Luc reporter vector, in the presence of *FoxO1* effector vector alone. White bar, mock (no DNA); black bar, pCDNA3 basic vector (without an insert). Representative WBs of HMGA1 in each condition are shown. β-actin, control of protein loading. Cropped blots are shown in the figures. Full-length WBs are presented in Supplementary Fig. S1.

To clarify whether HMGA1/FoxO1 interaction had a functional implication in the transcriptional activity of *IGFBP1* promoter, HEK-293 cells, which barely express HMGA1, were transfected transiently with an *IGFBP1* luciferase reporter construct (*IGFBP1*-Luc), in the presence of both HMGA1 and FoxO1 expression vectors. As shown in Fig. 4a, simultaneous overexpression of HMGA1 and FoxO1 in HEK-293 cells led to a significant increment in IGFBP1 Luc activity that exceeded that seen with either factor alone, resulting in synergistic activation of transcription. Higher amounts of HMGA1 failed to increase further *IGFBP1*-Luc activity probably due to the property of HMGA1 to bind non-specifically to the DNA when present at high concentrations^41^. As expected, treatment of transfected cells with insulin resulted in the reduction of reporter gene activity (Fig. 4a). To investigate further the functional significance of HMGA1-FoxO1 protein-protein interaction, we employed the *HMGA1m* expression vector coding for a mutant form of HMGA1, in which the three active serine phosphorylation sites (Ser98, Ser101 and Ser102) at the C-terminus of the HMGA1 protein were mutated to nonphosphorylatable alanines^23^. As previously reported, insulin elicited no effect on this mutant protein, which remained bound to DNA, thus demonstrating that phosphorylation at these sites is essential for regulating HMGA1 activity by insulin^23^. Consistently with this, when both *HMGA1m* and *FoxO1* expression vectors were simultaneously cotransfected with the *IGFBP1*-Luc reporter plasmid in HEK-293 cells, an increase in reporter activity was detected and this activity was maintained even after insulin treatment (Fig. 4a). Conversely, when transfection with *FoxO1* expression vector was preceded by siRNA-induced silencing of HMGA1 expression in HepG2 cells, a cell line naturally expressing HMGA1, *IGFBP1*-Luc activity was significantly reduced and this reduction was more pronounced in cells not exposed to *HMGA1* siRNA but treated with insulin (Fig. 4b). Interestingly, and in line with our previous original findings that HMGA1 may represent a critical nuclear mediator of insulin action^23^, insulin was ineffective in lowering *IGFBP1*-Luc activity in *HMGA1* siRNA-treated HepG2 cells (Fig. 4b).

### Dynamic interaction of HMGA1 with FoxO1

**Figure 5:**
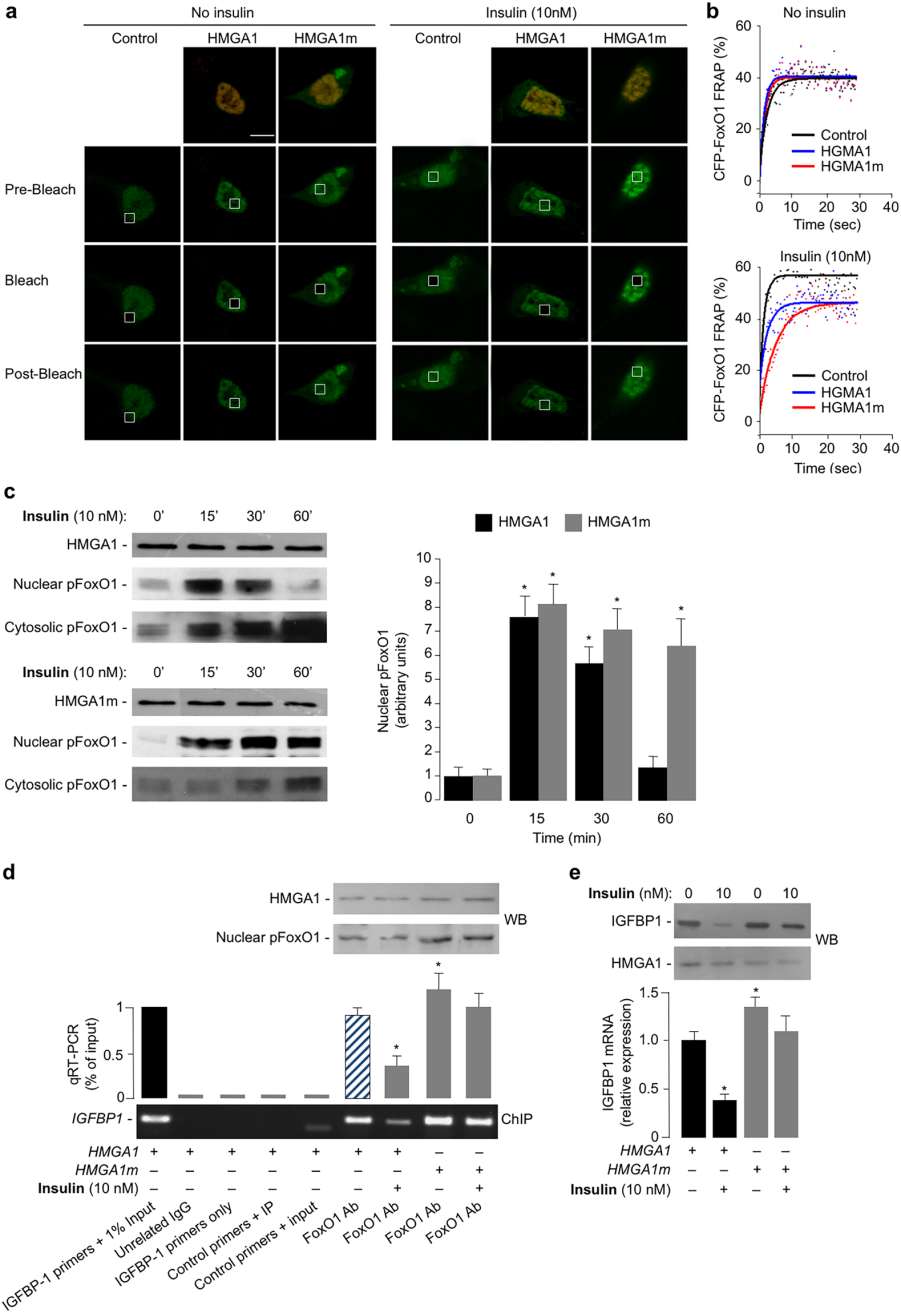
Insulin-induced HMGA1/FoxO1 phosphorylation and their kinetics. (**a**) Time lapse imaging of the intranuclear distribution of CFP-FoxO1 in HEK-293 cells, either untransfected (control), or transfected with *HMGA1* or *HMGA1m*, after treatment (0 and 20 min) with insulin. Pictures are optical sections made with a confocal laser scanning microscope. Bars correspond to 2 µm. Representative images of CFP-FoxO1 in HEK-293 cells relative to the FRAP experiment reported in (**b**). (**b**) FRAP analysis of FoxO1 in HEK-293 cells overexpressing HMGA1 wild-type or mutant HMGA1m. FRAP curves of CFP-FoxO1 in HEK-293 cells, either untreated (left) or treated with 10 nM insulin for 20 min (right). FRAP curves were calculated as described in Methods. (**c**) Time-course of the effect of insulin on FoxO1 phosphorylation in HEK-293 cells overexpressing HMGA1 (left) or HMGA1m (right). Representative WBs of HMGA1 and nuclear and cytosolic phosphorylated pFoxO1 are shown in both conditions. A comparison of the nuclear phosphorylated form of FoxO1 in cells overexpressing either HMGA1 (black bars) or HMGA1m (gray bars), under insulin treatment, is shown in bar graphs. Data are shown as the means ± s.e.m. of three separate experiments. Cropped blots are shown in the figure. Full-length WBs are presented in Supplementary Fig. S1. *P < 0.05 *vs* basal untreated cells (0 min). (**d**) Occupancy of the *IGFBP1* gene promoter by FoxO1, as measured by ChIP in HEK-293 cells transfected with either *HMGA1* or *HMGA1m* expression vectors, in the absence (−) or presence (+) of insulin. A representative assay is presented. qRT-PCR of ChIP-ed samples is shown in each condition. Cropped gel is shown in the figure. Full-length ChIP is presented in Supplementary Fig. S1. *P < 0.05 *vs* insulin untreated cells transfected with wild-type *HMGA1* effector plasmid (slashed bar). Immunoblots for the expression of nuclear HMGA1 and pFoxO1 in each condition are shown in the autoradiograms. Cropped blots are shown in the figure. Full-length WBs are presented in Supplementary Fig. S1. (**e**) mRNA and protein expression levels of IGFBP1 and HMGA1 in HEK-293 cells transfected with either *HMGA1* or *HMGA1m* expression vector in the absence (−) or presence (+) of insulin. Representative WBs are shown. Data are means ± s.e.m. of three separate experiments, each in triplicate. *P < 0.05 *vs* insulin untreated (HMGA1) cells. Cropped blots are shown in the figure. Full-length WBs are presented in Supplementary Fig. S1.

We next sought to investigate the dynamic interaction of HMGA1 and FoxO1 proteins in living cells. To this end, FRAP experiments were carried out in cells transfected with the CFP-tagged FoxO1 construct. By bleaching CFP-FoxO1 fluorescence in a defined nuclear region, we determined the time taken for fluorescence to recover by time-lapse confocal microscopy (Fig. 5a). Taking into account that the fluorescence recovery is dependent on the movement of unbleached CFP-FoxO1 molecules from the surrounding environment into the bleached region, it was then possible to measure the mobility of FoxO1 nuclear protein in CFP-FoxO1-expressing cells. In serum-starved HEK-293 cells, the fluorescence recovery of nuclear CFP-FoxO1 was incomplete (40% recovery), with a recovery time (t_1/2_) of 1.26 s, indicating that a significant fraction (~60%) of the CFP-FoxO1 fluorescent protein was bound to an immobile element within heterochromatic clusters (Fig. 5b). This observation fitted well with the notion that, in basal (starved) conditions, FoxO1 preferentially bound to DNA. Overexpression of either HMGA1 or HMGA1m in HEK-293 cells did not affect neither the fraction of immobile CFP-FoxO1 molecules (*P* = 0.28 and *P* = 0.71, respectively), nor the time of recovery for CFP-FoxO1 (*P* = 0.32 and *P* = 0.38, respectively) (Fig. 5b). After insulin treatment, the fluorescence recovery of CFP-FoxO1 was up to ~60%, with a shorter recovery time (t_1/2_ 0.91 s), as compared to unstimulated cells (*P* < 0.001), thus indicative of an increased nuclear mobility of the CFP-tagged FoxO1 protein (Fig. 5b), which is consistent with the insulin-induced nucleocytoplasmic shuttling of FoxO1. Overexpression of HMGA1 in HEK-293 cells under insulin treatment reduced the immobile fraction of CFP-FoxO1 to 46.7%, suggesting an increasing number of CFP-FoxO1 molecules being tethered within heterochromatic clusters, whereas similar kinetics of dissociation of CFP-FoxO1 from endogenous and over-expressed HMGA1 were observed (FRAP recovery time: t_1/2_ = 1.65 s) (Fig. 5b). Instead, in cells overexpressing the mutant form of HMGA1 (HMGA1m), the recovery time of CFP-FoxO1 was 3.37 s, significantly higher (*P* < 0.01) with respect to control cells expressing the wild-type HMGA1 protein, thus indicating a reduced mobility of FoxO1, and an impaired nucleocytoplasmic shuttling of phosphorylated FoxO1 (Fig. 5b). Increased nuclear retention of phosphorylated FoxO1 was confirmed in time course experiments of FoxO1 protein phosphorylation in cytosolic and nuclear fractions of HEK-293 cells overexpressing either the wild-type or the mutant HMGA1m protein, which underwent insulin treatment (Fig. 5c). These data were further substantiated by ChIP assays coupled with qRT-PCR of ChIP-ed samples, showing that binding of FoxO1 to the endogenous *IGFBP1* chromosomal locus was increased in HEK-293 cells overexpressing HMGA1m, in which the nuclear levels of pFoxO1 were higher with respect to cells overexpressing the wild-type form of HMGA1 (Fig. 5d). In line with this, as shown in Fig. 5e, IGFBP1 mRNA and protein levels were lower in insulin-treated HEK-293 cells overexpressing HMGA1 wild-type and higher in cells overexpressing the mutant HMGA1m protein, with these latter cells turning refractory to insulin with regard to the inhibition of IGFBP1.

Thus, taken togheter, these findings well support the functional relevance of HMGA1-FoxO1 interaction and provide evidence for the involvement of HMGA1 in insulin-mediated nucleocytoplasmic shuttling of FoxO1 protein.

### FoxO1 and IGFBP1 expression in primary hepatocytes from normal and *Hmga1-*deficient mice

**Figure 6:**
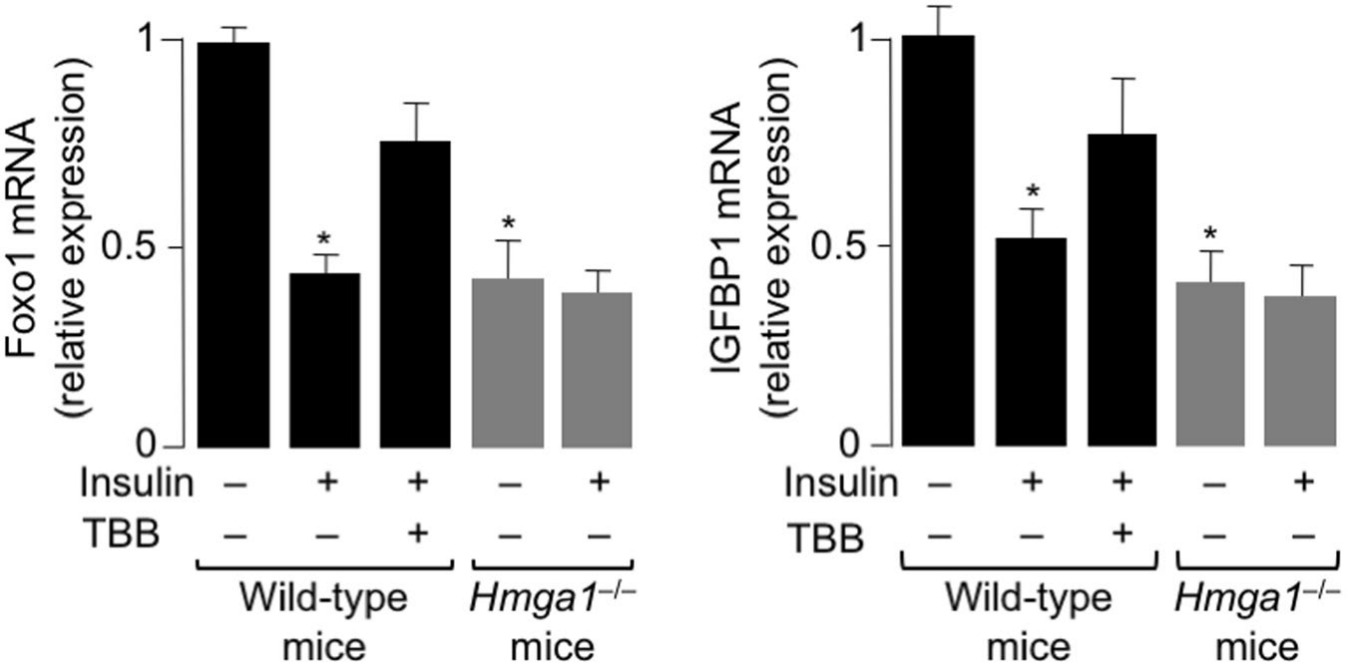
*FoxO1* and *IGFBP1* gene expression in primary cultured cells from normal and *Hmga1*-deficient mice. *FoxO1* and *Igfbp1* mRNA in primary cultured hepatocytes from wild-type (black bars) and *Hmga1*^−/−^ (gray bars) mice, either untreated or treated with insulin (10 nM), in the presence or absence of the protein kinase CK2 inkibitor TBB. Data are shown as the means ± s.e.m. of five independent experiments. *P < 0.05 *vs* untreated wild-type cells, in each assay.

To further explore the functional significance of HMGA1-FoxO1 interaction in nuclear insulin signaling, we then performed experiments in primary cultured hepatocytes from both wild-type and *Hmga1*^−/−^ mice. In these experiments, mRNA expression of FoxO1 and *Igfbp1* was measured in primary cultured cells that were pretreated with the casein kinase (CK) 2 inhibitor 4,5,6,7-tetrabromo-1H-benzimidazole (TBB), which selectively blocks insulin-induced HMGA1 phosphorylation, and the subsequent detachment of HMGA1 from DNA^23^, without affecting FoxO1, whose phosphorylation is, instead, dependent on CK1 and several other protein kinases that alter subcellular location of FoxO1, DNA binding and transcriptional activity^42–45^. Consistent with previous observations from our group^23,40,46^, mRNA expression of FoxO1 and Igfbp1 was lower in primary cultured *Hmga1*^−/−^ hepatocytes than in wild-type hepatocytes (Fig. 6). Following insulin treatment, the expression of mRNA for both FoxO1 and Igfbp1 decreased to 40–50% of control in cells from wild-type mice, whereas no changes in mRNA were observed in insulin-treated cells from mutant animals. Moreover, pretreatment of wild-type hepatocytes with TBB substantially attenuated the cellular response to insulin, with a less pronounced reduction in mRNA levels (Fig. 6), thereby indicating that the insulin’s effect on these two genes is mediated by HMGA1, and that functional cooperation between HMGA1 and FoxO1 may indeed constitute an essential step in nuclear insulin action.

### FoxO1 *in vivo*, in normal and *Hmga1* mutant mice

**Figure 7:**
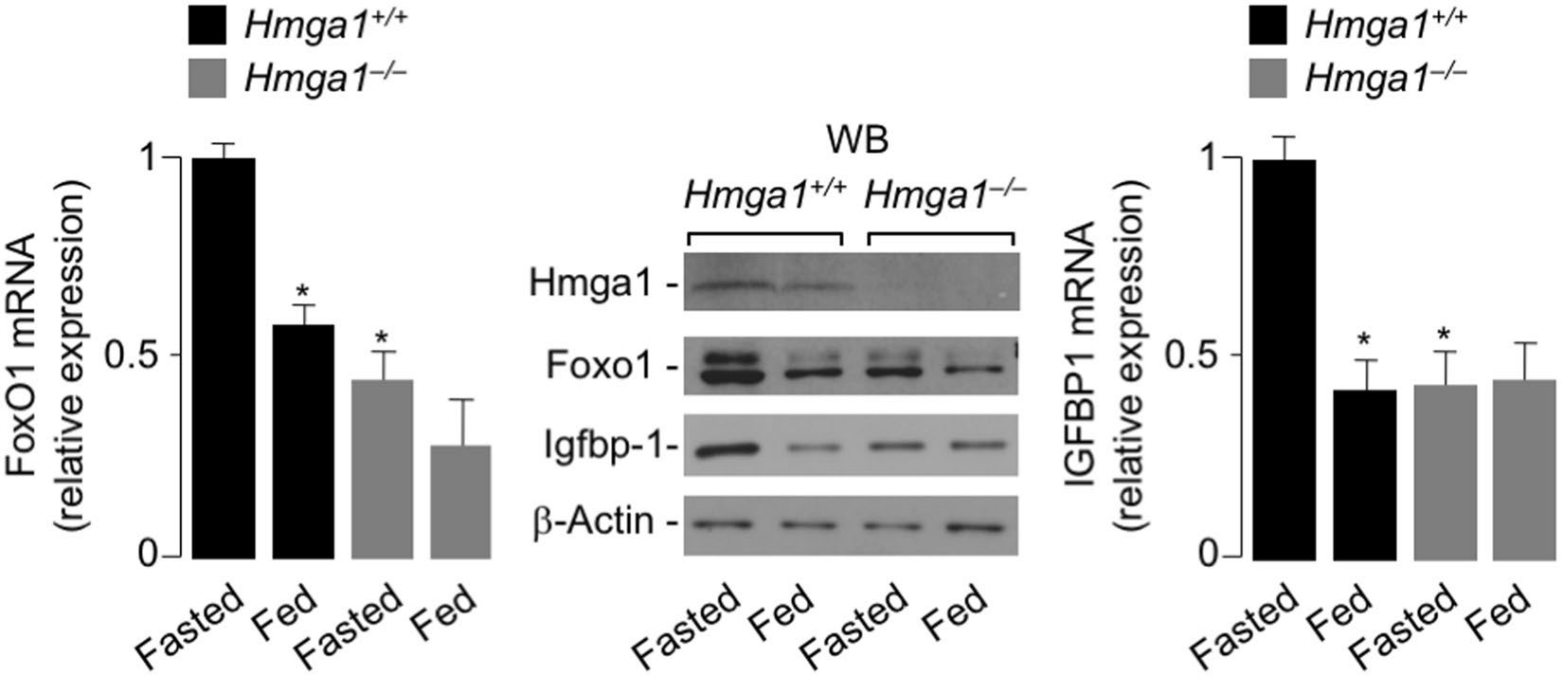
*FoxO1* and *IGFBP1* expression in liver from normal and *Hmga1*-deficient mice. Liver *FoxO1* and *Igfbp1* mRNA and protein levels were assayed in *Hmga1* wild-type (*Hmga1*^+/+^) and knockout (*Hmga1*^−/−^) 12 h-fasted mice (*n* = 6), and mice refed for 4 h (*n* = 6) with a high carbohydrate meal after a 12-h fast. mRNA was measured by qRT-PCR and normalized to *RPS9* mRNA abundance. *P < 0.05 *vs* fasted mice. Representative WBs of Hmga1, FoxO1, and Igfbp1 in both genotypes are shown in the autoradiograms. β-Actin, control of protein loading. Cropped blots are shown in the figure. Full-length WBs are presented in Supplementary Fig. S1.

As a further step toward understanding the relationship between HMGA1 and FoxO1, we extended the above *in vitro* studies to studies *in vivo*, in wild-type (*Hmga1*^+/+^) and *Hmga1*^−/−^ mice. As shown in Fig. 7, mRNA and protein abundances for FoxO1 were reduced in liver from 12 h-fasted *Hmga1*^−/−^ mice, compared with wild-type animals. After refeeding, when the insulin receptor signaling is reactivated, both FoxO1 mRNA and protein were significantly reduced in liver from wild-type animals, while no difference on these parameters was observed in *Hmga1*^−/−^ mice (Fig. 7), thereby confirming not only that HMGA1 is necessary for FoxO1 expression *in vivo*, in whole animals, but also that insulin-triggered FoxO1 downregulation is mediated by HMGA1. Similar results were obtained with mRNA and protein expression of Igfbp1 in liver from wild-type and *Hmga1*^−/−^ mice under the same experimental conditions than those used for FoxO1 expression (Fig. 7), thus supporting the role of HMGA1 in the transcriptional regulation of IGFBP1 through the insulin/FoxO1 pathway.

**Figure 8:**
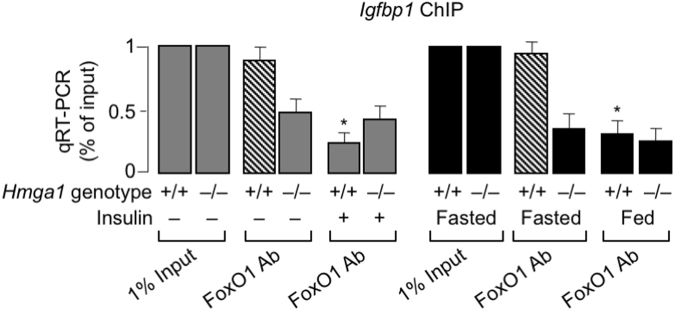
*FoxO1* occupancy at the endogenous *IGFBP1* locus *in vivo*, *in* normal and *Hmga1* mutant mice. Occupancy of the *Igfbp1* gene promoter by FoxO1 was measured by ChIP with anti-FoxO1 specific antibody (Ab) in liver from wild-type (+/+) and *Hmga1* mutant (−/−) mice, either untreated or treated with insulin (1 U/kg bw), or under fasting/fed conditions. Data are the mean values ± s.e.m. for three animals per genotype. *P < 0.05 *vs* control (slashed bar in each condition).

**Figure 9:**
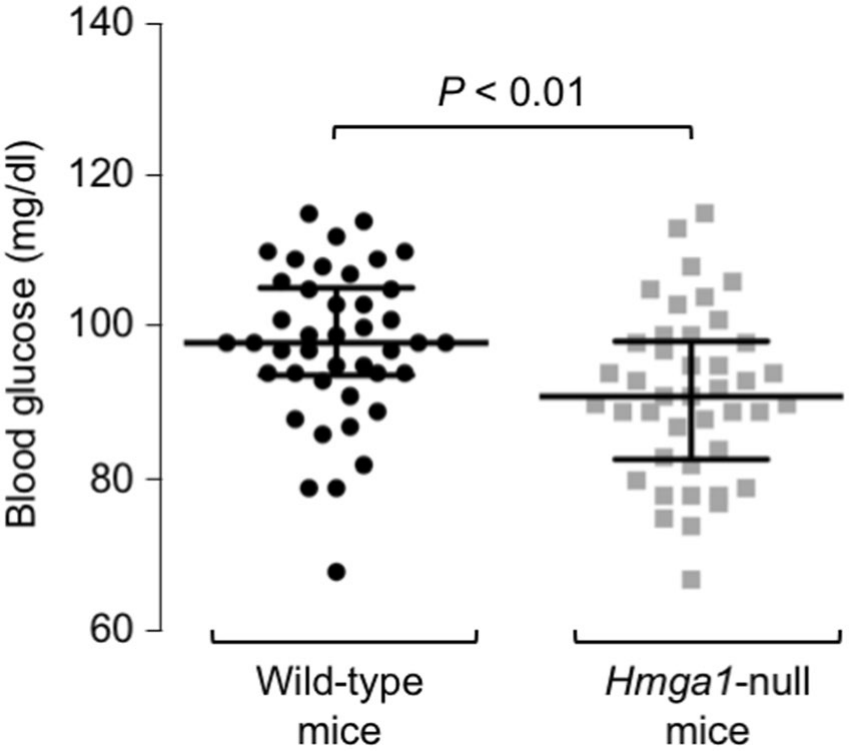
Fasting blood glucose values in wild-type and *Hmga1*-null mice. Levels of glucose were measured using a Glucocard glucometer (Menarini Diagnostics) from a blood drop collected from the tail tip of 12-h fasted conscious mice (*n* = 42 for each group). Bars indicate the median and interquartile range. The non-parametric Mann-Whitney test was applied for comparing the two groups.

The above results were confirmed by studies examining FoxO1 DNA-binding activity *in vivo*, in both wild-type and *Hmga1*^−/−^ mice. As measured by ChIP in whole liver tissue, and subsequent qRT-PCR of ChIP-ed samples, binding of the *Igfbp1* gene promoter by endogenous FoxO1 was reduced in *Hmga1*^−/−^ mice compared to *Hmga1*^+/+^ animals (Fig. 8). After insulin injection in living mice, binding of FoxO1 to DNA was significantly decreased in wild-type mice, while no differences were observed in *Hmga1*^−/−^ mice (Fig. 8). The same results were replicated in mice after meal ingestion, when endogenous insulin levels are expected to increase. As shown in Fig. 8, binding of FoxO1 to the *Igfbp1* locus was high in wild-type mice under fasting conditions when nutrients are limited, serum insulin is decreased and binding of HMGA1 to DNA is preferentially increased. Conversely, FoxO1-DNA interaction promptly decreased in wild-type mice after refeeding, when serum insulin increases, insulin signaling is reactivated and both FoxO1 and HMGA1 become phosphorylated (Fig. 8). In fasted HMGA1^−/−^ mice, binding of FoxO1 to the *Igfbp1* promoter was lower, compared to that in wild-type mice, but in contrast to normal animals, no change in FoxO1-DNA interaction was detected upon refeeding of mutant animals (Fig. 8), thus confirming that HMGA1 is indeed required for the functional regulation of FoxO1 by insulin *in vivo*. Consistent with the above observations, fasting glycemia was significantly lower in *Hmga1*-null mice, compared to wild-type animals (Fig. 9), confirming the results obtained in previous studies using a large number of both wild-type and *Hmga1*-mutant mice^40,46,47^.

## Discussion

As a member of the forkead family of transcription factors, FoxO1 regulates the transcription of a wide variety of genes involved in fundamental biological processes, such as cell growth and differentiation, DNA repair and apoptosis, inflammation and immune response^1,2^. Also, FoxO1 is a critical regulator of insulin signal transduction and energy metabolism^2,3^. In this last context, cytoplasm retention of FoxO1 via insulin-induced phosphorylation is considered to be a mechanism of insulin-mediated gene expression and regulation^3^. Dysregulation of FoxO1 at this level has been linked to metabolic abnormalities, including insulin resistance and type 2 diabetes^35^. Therefore, the observation that an interplay among HMGA1 and FoxO1 can be a component of the insulin/FoxO1 signaling pathway constitutes a novel point of the present study, which may help in understanding the molecular basis of certain disorders where insulin action becomes compromised (e.g. obesity, type 2 diabetes and other insulin-resistant conditions).

For the first time in the present work, we demonstrate that HMGA1 physically and functionally interacts with FoxO1, thereby regulating both FoxO1-DNA binding and insulin-mediated *FoxO1* gene transcription. Although a direct interaction between FoxO1 and HMGA1 has been observed in our study, in the absence of DNA, we show that binding of HMGA1 to the insulin target gene *IGFBP1* is necessary for FoxO1-DNA binding and transcriptional activity, and this may constitute an important requisite for the functional regulation of FoxO1 by insulin. In fact, when the ability of HMGA1 to bind DNA was prevented by distamycin A *in vitro*, in cultured cells, binding of FoxO1 to target DNA was markedly reduced, endogenous FoxO1-dependent gene expression was decreased, and insulin failed to downregulate FoxO1-DNA binding. Conversely, FoxO1-DNA binding and transcriptional activity were increased in cells overexpressing the mutant HMGA1m lacking the three serine phosphorylation sites responsive to insulin, in which, instead, the phosphorylation capability of FoxO1 by the PI3K/Akt pathway was not modified. Consistent with our results supporting a role for HMGA1 in nuclear FoxO1 mobility, nucleocytoplasmic shuttling of insulin-activated FoxO1 was impaired in cells overexpressing the mutant HMGA1m, whereas the nuclear trapping of FoxO1 was increased, thus providing compelling evidence that HMGA1 is indeed an important factor in modulating FoxO1 activity in the context of insulin receptor signaling and that the dynamic interaction between HMGA1 and FoxO1 plays a critical role in the control of insulin-mediated gene expression. In this regard, we previously reported that the counter-regulatory hormone glucagon, which acts in opposition to insulin to maintain fasting euglycemia, upregulated HMGA1 expression via the cAMP pathway, both *in vitro* and *in vivo* in whole mice^47,48^. Consistent with these previous findings, and with the observation that HMGA1 activates *FoxO1* gene expression^46^, upregulation of FoxO1 via the glucagon-cAMP-PKA signaling has been reported in liver of fasting mice to maintain fasting euglycemia^49^. Based on our findings here, it is likely that upregulation of HMGA1 during fasting (when glucagon peaks) may contribute to the maintenance of fasting euglycemia through two distinct but converging mechanisms: by increasing FoxO1 expression in response to glucagon, and by directly and dinamically interacting with FoxO1, thus playing an important role in fine-tuning the activation of FoxO1’s transcriptional activity. Exactly the opposite after meal, when insulin peaks in response to high glucose availability and glucagon is suppressed. In this metabolic setting, functional inactivation of HMGA1 following insulin-induced HMGA1 protein phosphorylation, by causing the detachment of FoxO1 from DNA and its inactivation by nuclear exclusion, represses hepatic gluconeogenesis, thereby contributing to the maintenance of postprandial glucose homeostasis.

The functional affinity between HMGA1 and FoxO1 is also underlined by the fact that other than phosphorylation, both proteins are also post-translationally regulated by the same histone acetyltransferase CBP (CREB-binding protein), which was found to catalyze the acetylation of both HMGA1 and FoxO1, with subsequent inactivation of transcriptional activity of both nuclear factors^50,51^. On the other hand, distinct roles of HMGA1 and FoxO1 in the regulation of pancreatic beta-cell function and insulin production have been reported by us and others^25,52,53^. However, it cannot be excluded the possibility that functional interaction of HMGA1 with FoxO1 may be cell specifically regulated by additional factors, so that cooperation between HMGA1 and FoxO1 in the transcriptional regulation of FoxO1-target genes is essential in some cell types, but not in others.

The possibility for a functional relationship between HMGA1 and FoxO1 is further supported by some metabolic similarities between *Hmga1*-deficient mice and liver-specific FoxO1-deficient mice, including insulin hypersensitivity of peripheral tissues following insulin tolerance test, and lower fasting plasma glucose levels as compared with wild-type animals^54–60^. Similarities in body weight and body composition profiles are also found in both knockout mouse models, in which no changes were observed in these parameters among normal and mutant mice^56,61,62^. Also, similarly to what was observed before, in mice lacking liver FoxO1^56^, a trend in increasing serum cholesterol levels was seen in *Hmga1*-knockout mice compared with controls, with a trend in reduction of triglycerides (unpublished data). Concerning this last point, it has to be admitted that conflicting results have been reported in liver-specific FoxO1-deficient mice, in which plasma triglycerides were either unaffected^56,60,63^ or increased^64^.

In toto, these findings establish HMGA1 as a critical element in the functional activity of FoxO1 transcription factor. Also, the data indicate that cross-talking between HMGA1 and FoxO1 is critical in relaying insulin signals down to the DNA, thereby ensuring insulin’s transcriptional regulation of glucose metabolism and homeostasis. Understanding this cross-talk is interesting from both biological and mechanistic standpoints and might be useful in understanding the molecular basis of disease states that arise from alterations in nuclear insulin signaling.

## Methods

### Ethics statement

The study was approved by the local ethics committee, Regione Calabria Comitato Etico Sezione Area Centro (protocol registry n. 116 of May 14, 2015), and the methods were performed in accordance with approved guidelines. All animal work was performed using approved animal protocols, according to relevant institutional guidelines for the care of laboratory animals (directive 86/609/ECC, European Community Council).

### Cell cultures and nuclear extracts

Human embryonic kidney 293 (HEK-293) cells and HepG2 human hepatoma cells were cultured in DMEM and RPMI 1640 medium, respectively, supplemented with 10% fetal bovine serum (FBS) (Gibco Laboratories), 2 mM glutamine, penicillin (100 U/ml), and streptomycin (100 µg/ml) in a humidified 5% CO_2_ atmosphere at 37 °C. Cytoplasmic and nuclear protein extracts were prepared as previously^24,65^ and final protein concentration in the extracts was determined by the modified Bradford method (Bio-Rad Laboratories)^66^.

### Glutathione S-transferase (GST) pull-down assay and co-immunoprecipitation

^35^S-labeled FoxO1 protein was synthesized by using the TNT-T7 quick-coupled *in vitro* transcription/translation system (Promega). GST tagged human HMGA1 was obtained using the pcDNA1-GST/HMGA1 expression vector, a kind gift from D. Thanos (Institute of Molecular Biology, Genetics and Biotechnology, Athens, Greece)^24^. Covalent coupling of antibody to protein A-Sepharose (GE Healthcare) was performed as previously described^67^. Antibody-coupled protein A beads were washed twice in phosphate-buffered saline and used in immunoprecipitation studies. Briefly, aliquots of HepG2 cell nuclear extract or pure HMGA1 were incubated for 3 h with rotation at 4 °C with 10 µl of antibody coupled protein A beads. Beads were recovered by gentle centrifugation and washed three times with 500 µl of NETN wash buffer [50 mM Tris-HCl (pH 8.0), 0.1% NP-40, 150 mM NaCl, 1 mM EDTA] for 5 min. Protein was removed from the beads by boiling in sample buffer for 5 min and analyzed by SDS-PAGE and immunoblotting^67^. Antibodies used for these studies were as follows: anti-HMGA1^24^ and anti-FoxO1 (sc-11350) (Santa Cruz Biotechnology).

### Plasmid construction and transfections, Fluorescence Resonance Energy Transfer (FRET), Fluorescence Recovery After Photobleaching (FRAP) analysis and confocal microscopy

Recombinant Luc reporter construct containing the human *IGFBP1* gene promoter has been described previously^23^. The construct was transiently transfected into HEK-293 or HepG2 cells, using the LipofectAMINE 2000 reagent (Invitrogen Life Technology Corporation), in the presence or absence of effector vector for HMGA1 (HMGA1a isoform protein)^23^ and/or FoxO1^68^, or HMGA1am^23^, and Luc activity was assayed 48 h later in a luminometer (Turner Biosystems Inc.), using the dual-luciferase reporter assay system (Promega). siRNA targeted to human HMGA1 (sc-37115) was purchased from Santa Cruz Biotechnology, whereas nonspecific siRNA controls with a similar GC content was obtained from Dharmacon (GE Dharmacon). 100–200 pmol siRNA duplex was transfected into cells at 40–50% confluency. After knockdown for 72 h, cells were trypsinized, pooled, and resuspended for a second transfection using the same targeting siRNA. After an additional 72 h, cells were prepared for analysis. Renilla control vector served as an internal control of transfection efficiency, together with measurements of protein expression levels.

To produce YFP-HMGA1a and CFP-FoxO1 expression plasmids, the human HMGA1a ORF (NCBI Ref. Seq. NM_145899.2) was cloned into BamHI/XbaI sites of pYFP (Clontech) and the human FoxO1 ORF (NCBI Ref. Seq. NM_002015.3) was cloned into BamHI/XbaI sites of pCFP (Clontech). Confocal imaging was performed with Leica SP2 inverted confocal microscope (Leica Mycrosystems), using 63 × Apo PLA oil immersion objective (NA 1.4) and 476 nm laser line. A 1 mm^2^ squared ROI within nucleus in heterochromatic region was set up for bleaching, 4 mm^2^ ROI for bleaching correction, and additionally 4 mm^2^ control ROI outside of cell area was acquired for background subtraction. The laser intensity was set up at 15%. Ten images were taken before the bleach pulse (15 laser iterations with bleaching intensity output 100%) and 60 images were taken after the bleaching with image acquisition each 0.3 s with 15% laser transmission. All FRAP experiments were performed in temperature-controlled CO_2_ supplied chamber. All FRAP plots were generated from background subtracted images using the public domain software ImageJ as previously described^69^. Shortly, the signal I was calculated in the area of interest (AOI) and normalized to the change in the total fluorescence due to the bleach pulse and the imaging: I = (To/Io) * It/Tt, where To and Tt are the total cellular fluorescence in the pre-bleach and post-bleach images, respectively, whereas Io is the average intensity in the AOI in the pre-bleach image.

### Chromatin immunoprecipitation (ChIP)

ChIP assay was performed in cultured HepG2 and HEK-293 cells, either untreated or pretreated with siRNA targeting HMGA1, as described previously^23,70^. For *in vivo* ChIP, at the end of the indicated treatments, mice were killed by cervical dislocation, the liver was rapidly removed, prewarmed with PBS, and treated with 0.2% collagenase for 15 min. The liver was then diced, forced through a 60 µm stainless steel sieve, the hepatocytes were collected directly into DMEM containing 1% formaldehyde, and the formaldehyde-fixed DNA-protein complexes were immunoprecipitated with the anti-FoxO1 antibody sc-11350 from Santa Cruz Biotechnology. Sequence-specific primers for the *IGFBP1* gene promoter used for PCR amplification of ChIP-ed DNA (30 cycles), using PCR ready-to-go beads (Amersham Pharmacia Biotech): human *IGFBP1* (NT_007819) for 5′-CAGAAAGAGAAGCAATTCCG-3′, rev 5′-TACCAGCCAGACGCGAGCAA-3′; mouse *Igfbp1* (NT_039515) for 59-CCTGGGGAGGGAGAAACAACT-39, rev 59-GCAGTGTTCAATGCTCGCTGG-39. PCR products were electrophoretically resolved on 1.5% agarose gel and visualized by ethidium bromide staining.

### Immunoprecipitation and Western blot

A polyclonal-specific antibody against HMGA1^23^ was used to analyze HMGA1 protein expression in HEK-293 and HepG2 cells, and in liver nuclear extracts from normal and *Hmga1* mutant mice. An anti-FoxO1 antibody (Santa Cruz Biotechnology) was employed to measure total FoxO1 in both human cell lines and animal tissues. An anti phospho-FoxO1 (Thr24) (Cell Signaling Technology) was used to analyze the levels of pFoxO1 protein in both nuclear and cytoplasmatic extracts.

### Studies in animals

Male *Hmga1*-deficient mice and wild-type littermates aged 6–9 months were studied. The generation of these animals has been described previously^25^. We have thoroughly investigated male mice to exclude the known effects linked to estrogen cyclicity, thereby limiting variability^62^. *FoxO1* and *Igfbp1* mRNA and protein levels were analyzed in liver from age and weight matched 12-h fasted mice and after 4 h refeeding. ChIP of the *Igfbp1* gene was performed in liver from 12 h-fasted animals following intraperitoneal injection of insulin (1U/kg bw) or saline, and after fasting and refeeding conditions. Cryopreserved primary hepatocytes from *Hmga1*^−/−^ and wild-type mice were cultured as previously described^23^.

### Statistical analysis

The non-parametric Mann-Whitney test was used for comparisons of continuous variables. A significance level of 0.05 was set for a type I error in all analyses. All bar graph data shown represent mean ± standard error of the mean (s.e.m.). All data were analyzed with SPSS 20.0 software (SPSS Inc.).

### Data availability

The datasets generated during the current study are available from the corresponding author on reasonable request.

## Electronic supplementary material

Supplementary Fig. S1

## References

[1] Accili D, Arden KC (2004). FoxOs at the crossroads of cellular metabolism, differentiation, and transformation. Cell.

[2] Kousteni S (2012). FoxO1, the transcriptional chief of staff of energy metabolism. Bone.

[3] Lee S, Dong HH (2017). FoxO integration of insulin signaling with glucose and lipid metabolism. J. Endocrinol..

[4] Biggs WH, Meisenhelder J, Hunter T, Cavenee WK, Arden KC (1999). Protein kinase B/Akt-mediated phosphorylation promotes nuclear exclusion of the winged helix transcription factor FKHR1. Proc. Natl. Acad. Sci. USA.

[5] Rena G, Prescott AR, Guo S, Cohen P, Unterman TG (2001). Roles of the forkhead in rhabdomyosarcoma (FKHR) phosphorylation sites in regulating 14–3–3 binding, transactivation and nuclear targeting. Biochem. J..

[6] Zhang X (2002). Phosphorylation of serine 256 suppresses transactivation by FKHR (FOXO1) by multiple mechanisms. Direct and indirect effects on nuclear/cytoplasmic shuttling and DNA binding. J. Biol. Chem..

[7] Matsuzaki H, Daitoku H, Hatta M, Tanaka K, Fukamizu A (2003). Insulin-induced phosphorylation of FKHR (FOXO1) targets to proteosomal degradation. Proc. Natl. Acad. Sci. USA.

[8] Huang H, Tindall DJ (2011). Regulation of FOXO protein stability via ubiquitination and proteasome degradation. Biochim. Biophys. Acta.

[9] Jing E, Gesta S, Kahn CR (2007). SIRT2 regulates adipocyte differentiation through FoxO1 acetylation/deacetylation. Cell Metab..

[10] Durham SK (1999). FKHR binds the insulin response element in the insulin-like growth factor binding protein-1 promoter. Endocrinology.

[11] Hall RK (2000). Regulation of phosphoenolpyruvate carboxykinase and insulin-like growth factor-binding protein-1 gene expression by insulin. The role of winged helix/forkhead proteins. J. Biol. Chem..

[12] Schmoll D (2000). Regulation of glucose-6-phosphatase gene expression by protein kinase B alpha and the forkhead transcription factor FKHR. Evidence for insulin response unit-dependent and –independent effects of insulin on promoter activity. J. Biol. Chem..

[13] Puigserver P (2003). Insulin-regulated hepatic gluconeogenesis through FOXO1-PGC-1alpha interaction. Nature.

[14] Guo S (1999). Phosphorylation of serine 256 by protein kinase B disrupts transactivation by FKHR and mediates effects of insulin on insulin-like growth factor-binding protein-1 promoter activity through a conserved insulin response sequence. J. Biol. Chem..

[15] Nakae J, Park BC, Accili D (1999). Insulin stimulates phosphorylation of the forkhead transcription factor FKHR on serine 253 through a wortmannin-sensitive pathway. J. Biol. Chem..

[16] Rena G, Guo SS, Cichy C, Unterman TG, Cohen P (1999). Phosphorylation of the transcription factor forkhead family member FKHR by protein kinase B. J. Biol. Chem..

[17] Tang ED, Nunez G, Barr FG, Guan KL (1999). Negative regulation of the forkhead transcription factor FKHR by Akt. J. Biol. Chem..

[18] Nakae J, Kitamura T, Silver DL, Accili D (2001). The forkhead transcription factor Foxo1 (Fkhr) confers insulin sensitivity onto glucose-6-phosphatase expression. J. Clin. Invest..

[19] Bustin M, Reeves R (1996). High-mobility group proteins: architectural components that facilitate chromatin function. Prog. Nucleic Acid Res. Mol. Biol..

[20] Thanos D, Maniatis T (1995). Virus induction of human IFN beta gene expression requires the assembly of an enhanceosome. Cell.

[21] Reeves R (2001). Molecular biology of HMGA proteins: hubs of nuclear function. Gene.

[22] Cleynen I, Van de Ven WJ (2008). The HMGA proteins: a myriad of functions. Int. J. Oncol..

[23] Chiefari E (2012). HMGA1 is a novel downstream nuclear target of the insulin receptor signaling pathway. Sci. Rep..

[24] Foti D, Iuliano R, Chiefari E, Brunetti A (2003). A Nucleoprotein Complex Containing Sp1, C/EBPβ, and HMGI-Y Controls Human Insulin Receptor Gene Transcription. Mol. Cell. Biol..

[25] Foti D (2005). Lack of the architectural factor HMGA1 causes insulin resistance and diabetes in humans and mice. Nat. Med..

[26] Chiefari E (2013). A polymorphism of HMGA1 is associated with increased risk of metabolic syndrome and related components. Sci. Rep..

[27] Pullinger CR (2014). Evidence that an HMGA1 gene variant associates with type 2 diabetes, body mass index, and high-density lipoprotein cholesterol in a Hispanic-American population. Metab. Syndr. Relat. Disord..

[28] Bianco A (2015). The association between HMGA1 rs146052672 variant and type 2 diabetes: A transethnic meta–analysis. PLoS One.

[29] Arce–Cerezo A (2015). HMGA1 overexpression in adipose tissue impairs adipogenesis and prevents diet–induced obesity and insulin resistance. Sci. Rep..

[30] Kode A (2012). FoxO1 protein cooperates with ATF4 protein in osteoblasts to control glucose homeostasis. J. Biol. Chem..

[31] Nakae J (2012). Novel repressor regulates insulin sensitivity through interaction with Foxo1. EMBO J..

[32] Shats I (2013). FOXO transcription factors control E2F1 transcriptional specificity and apoptotic function. Cancer Res..

[33] Kim DY, Hwang I, Muller FL, Paik JH (2015). Functional regulation of FoxO1 in neural stem cell differentiation. Cell Death Differ..

[34] Puig O, Tjian R (2005). Transcriptional feedback control of insulin receptor by dFOXO/FOXO1. Genes Dev..

[35] Pajvani UB, Accili D (2015). The new biology of diabetes. Diabetologia.

[36] Daitoku H, Sakamaki J, Fukamizu A (2011). Regulation of FoxO transcription factors by acetylation and protein-protein interactions. Biochim. Biophys. Acta.

[37] Obsil T, Obsilova V (2011). Structural basis for DNA recognition by FOXO proteins. Biochim. Biophys. Acta.

[38] Calnan DR, Brunet A (2008). The FoxO code. Oncogene.

[39] Greco M (2014). Early effects of a hypocaloric, Mediterranean diet on laboratory parameters in obese individuals. Mediators Inflamm..

[40] Iiritano S (2012). The HMGA1-IGF-I/IGFBP system: a novel pathway for modulating glucose uptake. Mol. Endocrinol..

[41] Yie J, Liang S, Merika M, Thanos D (1997). Intra- and intermolecular cooperative binding of HMG I (Y) to the IFNβ promoter. Mol. Cell. Biol..

[42] Rena G (2002). Two novel phosphorylation sites on FKHR that are critical for its nuclear exclusion. EMBO J..

[43] Lee JW, Chen H, Pullikotil P, Quon MJ (2011). Protein kinase A-alpha directly phosphorylates FoxO1 in vascular endothelial cells to regulate expression of vascular cellular adhesion molecule-1 mRNA. J. Biol. Chem..

[44] van der Heide LP, Smidt MP (2005). Regulation of FoxO activity by CBP/p300-mediated acetylation. Trends Biochem. Sci..

[45] Huang H, Regan KM, Lou Z, Chen J, Tindall DJ (2006). CDK2-dependent phosphorylation of FOXO1 as an apoptotic response to DNA damage. Science.

[46] Arcidiacono B (2017). HMGA1 is a novel transcriptional regulator of the FoxO1 gene. Endocrine.

[47] Chiefari E (2009). The cAMP-HMGA1-RBP4 system: a novel biochemical pathway for modulating glucose homeostasis. BMC Biol..

[48] Bianconcini A (2009). Transcriptional activity of the murine retinol-binding protein gene is regulated by a multiprotein complex containing HMGA1, p54 nrb/NonO, protein-associated splicing factor (PSF) and steroidogenic factor 1 (SF1)/liver receptor homologue 1 (LRH-1). Int. J. Biochem. Cell Biol..

[49] Wondisford AR (2014). Control of Foxo1 gene expression by co-activator P300. J. Biol. Chem..

[50] Zhang Q, Wang Y (2008). High mobility group proteins and their post-translational modifications. Biochim. Biophys. Acta.

[51] Daitoku H (2004). Silent information regulator 2 potentiates Foxo1-mediated transcription through its deacetylase activity. Proc. Natl. Acad. Sci. USA.

[52] Arcidiacono B (2015). Cooperation between HMGA1, PDX–1 and MafA is essential for glucose–induced insulin transcription in pancreatic beta cells. Front. Endocrinol..

[53] Talchai C, Xuan S, Lin HV, Sussel L, Accili D (2012). Pancreatic beta cell dedifferentiation as a mechanism of diabetic beta cell failure. Cell.

[54] Altomonte J (2003). Inhibition of Foxo1 function is associated with improved fasting glycemia in diabetic mice. Am. J. Physiol. Endocrinol. Metab..

[55] Dong XC (2008). Inactivation of hepatic Foxo1 by insulin signaling is required for adaptive nutrient homeostasis and endocrine growth regulation. Cell Metab..

[56] Matsumoto M, Pocai A, Rossetti L, Depinho RA, Accili D (2007). Impaired regulation of hepatic glucose production in mice lacking the forkhead transcription factor Foxo1 in liver. Cell Metab..

[57] Samuel VT (2006). Targeting foxo1 in mice using antisense oligonucleotide improves hepatic and peripheral insulin action. Diabetes.

[58] Lu M (2012). Insulin regulates liver metabolism *in vivo* in the absence of hepatic Akt and Foxo1. Nat. Med..

[59] Xiong X, Tao R, DePinho RA, Dong XC (2013). Deletion of hepatic FoxO1/3/4 genes in mice significantly impacts on glucose metabolismthrough downregulation of gluconeogenesis and upregulation of glycolysis. PLoS ONE.

[60] Zhang W (2006). FoxO1 regulates multiple metabolic pathways in the liver: effects on gluconeogenic, glycolytic, and lipogenic gene expression. J. Biol. Chem..

[61] Fedele M (2006). Haploinsufficiency of the Hmga1 gene causes cardiac hypertrophy and myelo-lymphoproliferative disorders in mice. Cancer Res..

[62] O-Sullivan I (2015). FoxO1 integrates direct and indirect effects of insulin on hepatic glucose production and glucose utilization. Nat. Commun..

[63] Kamagate A (2008). FoxO1 mediates insulin-dependent regulation of hepatic VLDL production in mice. J. Clin. Invest..

[64] Haeusler RA, Pratt-Hyatt M, Welch CL, Klaassen CD, Accili D (2012). Impaired generation of 12-hydroxylated bile acids links hepatic insulin signaling with dyslipidemia. Cell Metab..

[65] Brunetti A (1993). Identification of unique nuclear regulatory proteins for the insulin receptor gene, which appear during myocyte and adipocyte differentiation. J. Clin. Invest..

[66] Brunetti A (1996). Human diabetes associated with defects in nuclear regulatory proteins for the insulin receptor gene. J. Clin. Invest..

[67] Paonessa F (2006). Activator protein-2 overexpression accounts for increased insulin receptor expression in human breast cancer. Cancer Res..

[68] Armoni M (2006). FOXO1 represses peroxisome proliferator-activated receptor-gamma1 and -gamma2 gene promoters in primary adipocytes. A novel paradigm to increase insulin sensitivity. J. Biol. Chem..

[69] McNally JC (2008). Quantitative FRAP in analysis of molecular binding dynamics *in vivo*. Methods Cell Biol..

[70] Costa V (2008). The insulin receptor: a new anticancer target for peroxisome proliferator-activated receptor-gamma (PPARgamma) and thiazolidinedione-PPARgamma agonists. Endocr. Relat. Cancer.

