# Supplementary material for: Cross-talk among HMGA1 and FoxO1 in control of nuclear insulin signaling
Source: Sci Rep. 2018 Jun 4;8:8540. doi: 10.1038/s41598-018-26968-3 (PMC5986867; doi:10.1038/s41598-018-26968-3)
Supplement: Supplementary file 1 — Supplementary Fig. S1 [file 41598_2018_26968_MOESM1_ESM.pdf]

Cross-talk among HMGA1 and FoxO1 in control of nuclear insulin signaling

Eusebio Chiefari, Biagio Arcidiacono, Camillo Palmieri, Domenica Maria Corigliano, Valeria Maria Morittu, Domenico Britti, Michal Armoni, Daniela Patrizia Foti, & Antonio Brunetti

Supplementary Information

Supplementary Fig. S1

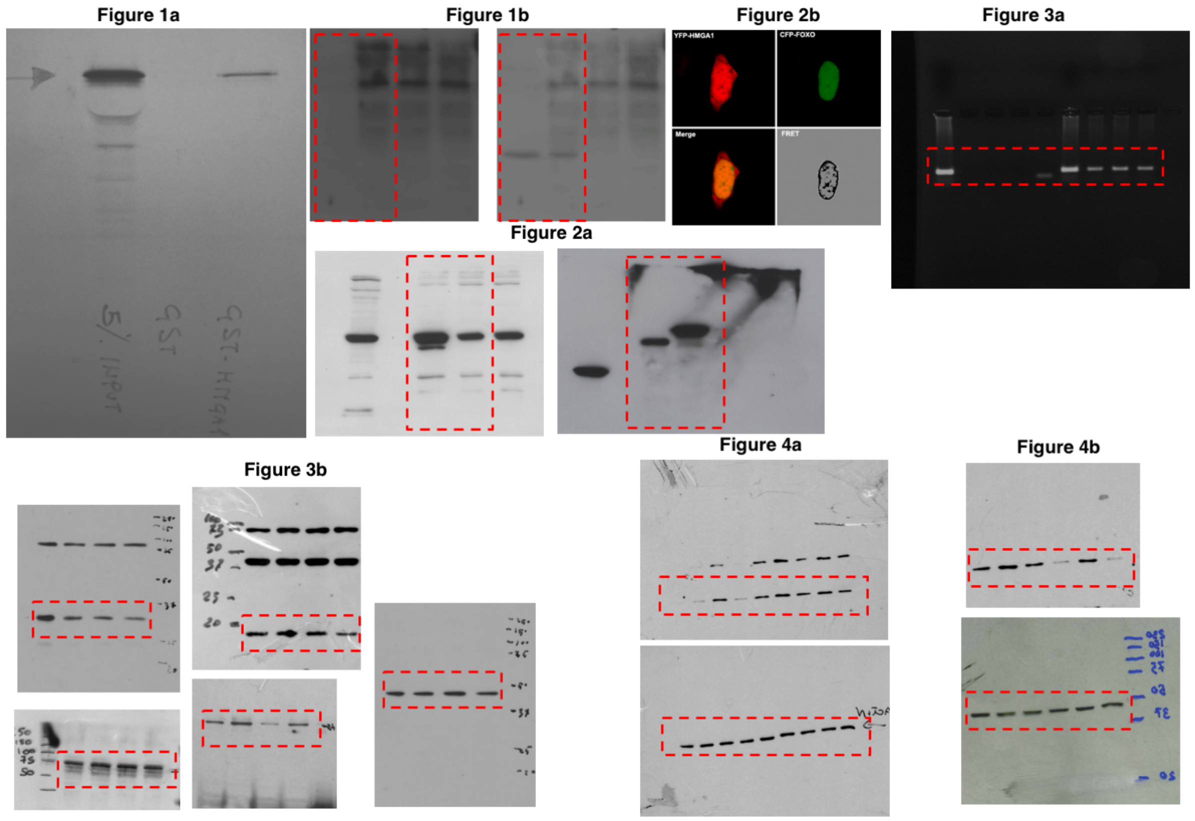

Figure 5a

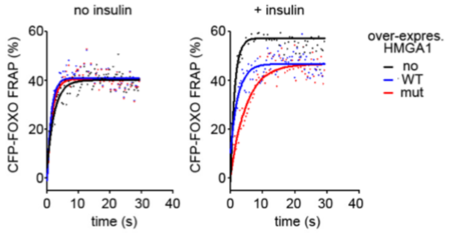

Figure 5b

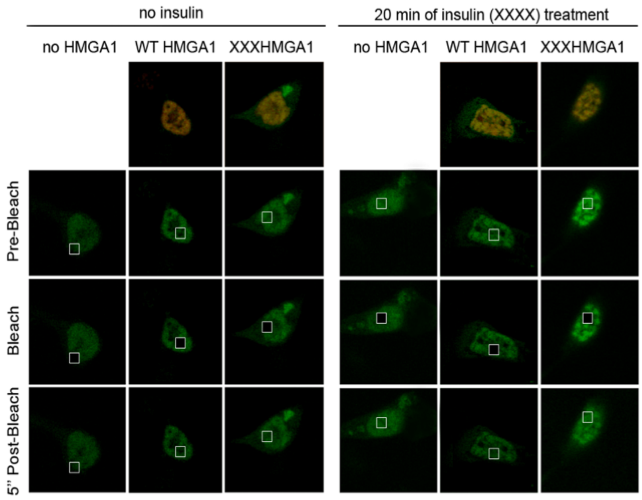

Figure 5c

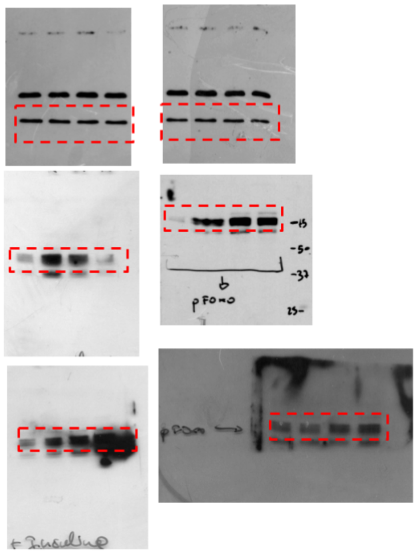

Figure 5d

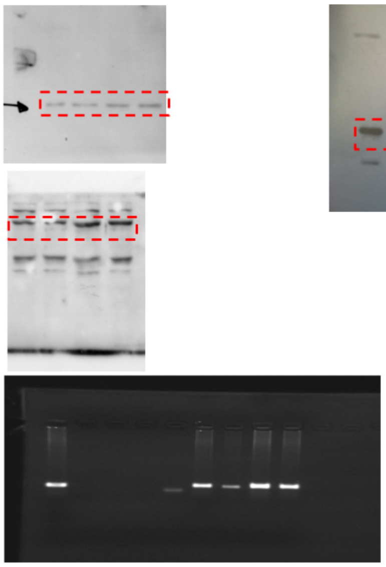

Figure 5e

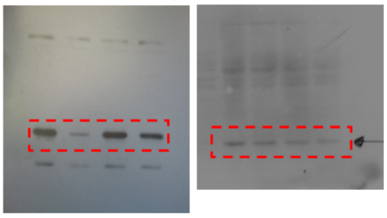

Figure 7

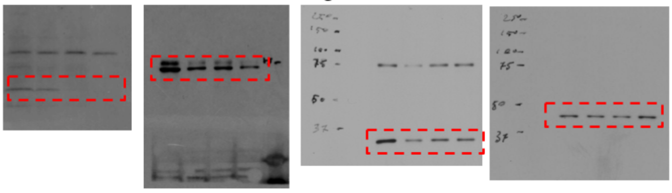

Supplementary Fig. S1: **Full-length blots and gels**

GST-Pull-down: Figure 1a;
Western blots: Figure 1b, FoxO1, HMGA1; Figure 2a, YFP-HMGA1, CFP-FoxO1, FoxO1; Figure 3b, IGFBP1, HMGA1, FoxO1, pFoxO1, β-actin; Figure 4a (HEK-293), HMGA1, β-actin; Figure 4b (HepG2), HMGA1, β-actin; Figure 5c, HMGA1, nuclear pFoxO1, cytosolic

pFoxO1, HMGA1m, nuclear pFoxO1, cytosolic pFoxO1; Figure 5d, HMGA1, nuclear pFoxO1; Figure 5e, IGFBP1, HMGA1; Figure 7, Hmga1, Foxo1, Igfbp1, β-actin.
ChIP: Figure 3a, Figure 5d.
